# The role of TGFbeta signals in Lead (Pb)‐induced Cerebral Amyloid Angiopathy

**DOI:** 10.1002/alz.086643

**Published:** 2025-01-03

**Authors:** Huiying Gu, Alexandria Luo, Wei Zheng, Yansheng Du

**Affiliations:** ^1^ Indiana University School of Medicine, Indianapolis, IN USA; ^2^ Purdue University School of Health Sciences, West Lafayette, IN USA

## Abstract

**Background:**

Mounting evidence suggests that acute and past exposure to the environmental toxicant lead (Pb) results in longitudinal decline in cognitive function and brain atrophy. In animals, chronic Pb exposure can increase brain Aβ deposition. However, it remains unclear how Pb induces different natures of amyloid depositions and underlying mechanisms to contribute to the pathogenesis of AD and related dementia.

**Method:**

Female APP/PS1 mice at 8 weeks old were administered with either 50 mg/kg Pb‐acetate (PbAc) (i.e., 27 mg Pb/kg) via oral gavage or an equivalent molar concentration of Na‐acetate (NaAc) once daily for additional 3 days or 8 weeks, in the presence or absence of an PAI‐1 inhibitor (12mg/kg). Parenchymal plaques and vascular amyloid deposition were quantitated by double staining with Thioflavin S and anti‐collagen IV antibody. Brain sections were also stained with anti‐NeuN (for neurons), anti‐myelin basic protein (for myelination) and anti‐rabbit FITC (for reactive astrocytes) antibodies. Assays for perivascular drainage as well as in vitro vascular binding with Aβ and microglial endocytosis were also performed.

**Result:**

2‐month Pb exposure increased vascular Aβ deposits in neocortex of female APP/PS1 mice at the 4‐month of age by almost 300% (p<0.01). In contrast, Pb only increased parenchymal amyloid in the same brain areas by 86.7% (p<0.05). Demyelination, but not neuronal loss was observed in Pb‐treated AD mice that had significantly cognitive deficits detected by Y‐maze. Following Pb treatments, the ratios of Aβ1‐40/Aβ1‐42 in Pb‐treated groups increased to 0.58 ± 0.21 in the cortical parenchyma and 0.50 ± 0.10 in the brain vasculature, as compared to those in the control group (0.39 ± 0.09 and 0.11 ± 0.02). Additionally, TGF‐β1, Smad2, PAI‐1 and fibronectin were significantly induced in cerebrovasculature isolated from mice treated with 27 mg/kg of Pb for 3 days, accompanied by dramatic inhibition of perivascular drainage and vascular binding with Aβ1‐40. Furthermore, Pb exposure induced microglial TGF‐β1 and inhibited clearance of Aβ40 and LRP‐1 expression. Interestingly enough, all of these alterations induced by Pb exposure could be markedly ameliorated by a PAI‐1 inhibitor.

**Conclusion:**

TGF‐β signals play distinct roles in Pb‐induced amyloid pathology.

